# The Acute Phase Proteins Reaction in Children Suffering from Pseudocroup

**DOI:** 10.1155/2019/6518308

**Published:** 2019-03-26

**Authors:** Beata Pucher, Magdalena Sobieska, Michal Grzegorowski, Jaroslaw Szydlowski

**Affiliations:** ^1^Department of Pediatric Otolaryngology of Poznan University of Medical Sciences, 60-572 Poznan, Szpitalna 27/33, Poland; ^2^Department of Physiotherapy, Chair for Physiotherapy and Rehabilitation of Poznan University of Medical Sciences, 60-545 Poznan, 28 Czerwca 1956 r. 135/147, Poland

## Abstract

The aim of the study was to evaluate the inflammatory reaction in children with pseudocroup and compare it with other laryngological diseases according to the available literature data. The study group included 51 children hospitalized because of pseudocroup. The measurements of the acute phase proteins (APP), such as C-reactive protein (CRP), alpha-1-antitrypsin (AT), alpha-1-antichymotrypsin (ACT), alpha-1-acid glycoprotein (AGP), ceruloplasmin (Cp), transferrin (Tf), alpha-2-macroglobulin (A2M), and haptoglobin (Hp) were obtained at 3 time points. The glycosylation profiles of AGP, ACT, and Tf were completed. An increased AGP level was observed in girls. The AGP glycosylation revealed the advantage of the W0 variant over the W1 variant. W1 and W2 were decreased in boys. W3 emerged in boys. The Tf concentration and T4 variant were lower compared to the control group. The A2M level was lower after treatment. The Hp and AT levels were decreased a few weeks later. The ACT glycosylation revealed a decrease of the A4 variant in boys. In conclusion, the inflammatory reaction during pseudocroup was of low intensity. The APP glycosylation suggested a chronic process. In a follow-up investigation, no normalization of the parameters was noted, but signs of persistent inflammation were observed.

## 1. Introduction

Subglottic laryngitis (pseudocroup) is a respiratory illness characterized by inspiratory stridor and barking cough. These symptoms result from an inflammation in the larynx at the level of the subglottic region. It occurs mainly in infants and small children between 6 and 36 months of age, more frequently in boys. Parainfluenza virus type 1 is the most common cause of pseudocroup, especially during the fall and winter epidemics when it reaches the major incidence peak [1]. Although pseudocroup is usually a mild self-limited disease, it might be a potentially life-threatening condition demanding hospitalization and prompt management and airway preservation [2–5].

Different response mechanisms exist within the respiratory system. During the acute phase reaction (which is a part of the innate immune response), the basic levels of various proteins maintained by homeostatic mechanisms can change substantially. These changes are thought to contribute to host defense and other adaptive capabilities. Acute phase proteins (APP) are defined as those proteins whose serum concentrations increase or decrease by at least 25% during inflammation. Such proteins are called either positive or negative APP, respectively. The measurement of their serum levels is therefore used as a laboratory marker of the intensity of the inflammatory process. At least 40 different plasma proteins have been identified as APP. The liver is generally thought to be the main organ responsible for the secretion of all the APP into the blood as part of the systemic acute phase reaction. Several APP are widely used to monitor many diseases. Their assessment in peripheral blood represents some specificity and high sensitivity [6]. In humans, CRP is the most elevated APP in bacterial pneumonia but only a little increase of CRP is found in severe viral acute respiratory syndrome, while the greatest increase can be seen in other APP, for example, alpha-1-antitrypsin (AT) [7]. According to Schrodl et al. in 2016, the central and extrahepatic expression of several APP is a common characteristic of normal and injured tissues and the search for the tissue/disease-specific characteristics of the APP and their possible applicability in diagnostics is needed [6]. The APP vary during the course of an inflammatory condition, and the group analysis of the APP profile is more significant than measuring any single protein. Quantitative and qualitative changes in the concentration of acute phase proteins will hopefully allow assessing the course of infection in children with subglottic laryngitis, detecting the presence of bacterial coinfection before clinical symptoms appear, and monitoring the course of treatment.

### 1.1. The Aim of the Study

The aim of the study was to evaluate the character and the intensity of the inflammatory reaction in children with subglottic laryngitis and compare the results with other laryngological diseases according to the available literature data.

## 2. The Study Design

The study group included 51 children (10 girls and 41 boys, aged from 6 months to 7 years, mean 3.8 ± 2.5), hospitalized in the Pediatric Otolaryngology Department because of the subglottic laryngitis. Only children with symptoms requiring hospitalization, i.e., inspiratory dyspnea, stridor, and barking cough were qualified to the study group. The children with symptoms of acute lower respiratory tract infection were excluded from the study group. The results of the routine laboratory tests for the study group at the time of admission which were within normal limits are presented in Table 1.

The measurements of nonspecific parameters of acute phase response, such as C-reactive protein (CRP), alpha-1-antitrypsin (AT), alpha-1-antichymotrypsin (ACT), alpha-1-acid glycoprotein (AGP), ceruloplasmin (Cp), transferrin (Tf), alpha-2-macroglobulin (A2M), and haptoglobin (Hp), were obtained from each individual at 3 time points: admission (sample no. 1), after the termination of treatment (sample no. 2), and during the control visit (at least 3 weeks after the treatment; sample no. 3).

In the control group, which included 22 healthy children (10 girls and 12 boys, aged 3 to 6 years, mean 4.2 ± 1.1) from the kindergarten, the blood sample was obtained once during routine health checkup, and permission was obtained from the University Bioethics Committee.

For the determination of the levels of APP, the Laurell rocket immunoelectrophoresis method was used and the standard solution for human serum proteins and antibodies from Dakopatts A/S (Denmark) was applied. In order to obtain the qualitative analysis of APP, the glycosylation profiles of AGP, ACT, and Tf were performed with use of the Bøg-Hansen cross-affinity immunoelectrophoresis method modified by Mackiewicz and Mackiewicz [8].

The distribution of all interval variables was first checked by the Shapiro-Wilk test. As all the investigated variables showed distribution distinct from normal, median and quartiles were used for the characteristics and nonparametric tests were applied (the Mann–Whitney *U* test was used to compare the variables between the study group and control group, and the Wilcoxon test was used for the time changes). The significance level of *p* < 0.05 was chosen.

## 3. Results

### 3.1. The Clinical Presentation

In all children, a detailed ENT examination was performed at the time of the admission which confirmed the presence of pseudocroup and excluded other acute laryngeal and upper and lower inflammatory respiratory tract pathologies. The thorough history of the concomitant diseases was taken.

### 3.2. The Estimation of the Levels of the APP and Their Microheterogeneity in Children with Pseudocroup at Three Time Points

The mean value of AGP was higher after the termination of treatment (sample no. 2) when compared to the day of admission (sample no. 1). During the control visit (sample no. 3), its level was lower when compared to the control group value. These differences were not statistically significant. The AGP-RC factor (AGP reactivity coefficient with concanavalin) was higher at the beginning of the disease and after the end of treatment, and these differences were significantly important in sample no. 2 (*p* = 0.025). The qualitative assessment of the glycosylation profile of the AGP revealed the advantage of the W0 variant over the W1 variant. The concentration of W0 was lower in all three samples in comparison to the control group but notable only in sample no. 2 (*p* = 0.02). W1 was remarkably lower in all samples in comparison to the control group (in sample no. 1—0.003, in sample no. 2—0.01, and in sample no. 3—0.004, respectively), while W2 was significantly higher in all 3 samples related to the control group. The appearance of the W3 variant was noted at the beginning of the disease and at the end of the treatment.

The analysis of the ACT glycosylation profile revealed the higher concentration of A2 in comparison to A1 and also the appearance of the A5 variant which was statistically significant in samples 2 and 3.

The Tf concentration and its T4 variant was notably lower in all 3 samples when compared to the control group. The A2M level was lower right after the treatment, and the Hp and AT levels were decreased a few weeks after the treatment of pseudocroup. All the values (Tf, T4, Hp, and AT) were of statistical significance.

The results of the investigations of the APP and their glycosylation profiles for the control group and the study group at the 3 time points (statistically significant differences are marked in bold; the Mann–Whitney *U* test value and *p* value are given) are presented in Table 2.

The Wilcoxon test results of the three samples and the control group are presented in the Table 3 (with statistically significant differences marked in bold).

### 3.3. Analysis of the APP Serum Levels Varying on the Sex of the Study Group in Each Time Point (3 Samples)

The study group (*n* = 51) consisted of 41 boys and 10 girls. The prevalence of pseudocroup was significantly more frequent in boys, with a ratio of 4.1 : 1.

An increased level of AGP was observed in girls with statistical significance at admission (sample no. 1) when compared to the control group. The W1 and W2 variants were notably decreased in boys in all samples (at admission, after the treatment, and during the control visit). Also, the W3 variant significantly emerged in boys in sample nos. 1 and 2 (*p* = 0.002 and *p* = 0.003, respectively).

The ACT glycosylation profile revealed a noteworthy decrease of the A4 variant in boys in all three samples.

The Tf levels were much lower in girls than in boys in all three samples, but the differences in its level between the 3 samples were statistically significant in boys only. The T4 variant was higher in girls with statistical significance in all three time points of the examination.

## 4. Discussion

The evaluation of the APP concentrations showed insignificantly higher values in comparison to control group in the first and second time points, which can be regarded as the acute inflammation of low density, typical in diseases of viral etiology. This was indicated by both undetectable CRP and slightly increased concentrations of AGP and ACT. Also, the results of the routine laboratory tests did not show any major changes towards reference values. Similarly, Wander et al. reported in 2014 about marginally elevated AGP and CRP in children between 3 and 5 years of age during an acute viral infection disease (with symptoms like vomiting, fever, and diarrhea) [9]. The AGP glycosylation profile was altered towards higher reactivity with ConA in the study group in comparison to the control group, and it was typical for acute inflammation, as well as for nonbacterial etiology and low intensity inflammation. These results differed significantly from the AGP glycosylation profile observed in children with bacterial diseases or posttraumatic conditions or after surgical procedures [10–13].

The alterations in ACT concentration compared to the control group and in children during pseudocroup treatment were insignificant. A slight lowering of ConA reactivity was observed for ACT in the study group, but the difference towards the control group was not significant. This indicates the absence of an intensified inflammatory process, and it can be concluded that inflammatory changes during subglottic laryngitis are not accompanied by necrotic changes, observed for example in children with chronic tonsillitis [14].

In the first time point (sample no. 1), as well as in subsequent time points of the course of treatment, a slight decrease in Tf concentration was observed in comparison to the control group. Despite the viral etiology, inflammation activates the mechanisms that limit the availability of iron, by withdrawing Tf from the blood circulation; this is mainly the T3 variant which easily binds and releases iron and seems to correspond to apotransferrin. On the other hand, the percentage of the T1 variant, which binds iron permanently, is higher [15]. In 2007, Formanowicz et al. obtained similar results in adult patients with the final stage of renal disease treated with hemodialysis [16]. No similar data is available for children. The decrease of the total Tf concentration and T3 variant and the increase in the percentage share of the T1, T2, and T4 variants in the total amount of Tf were achieved, and these are characteristic of a chronic inflammatory process [16]. The lower values of Tf might reflect a much more intense inflammation in girls, which could indicate better disease control.

A temporary decrease in A2M concentration was also observed in the second study compared to the control group. A2M has many diversified and complex functions, but it is primarily known by its ability to inhibit a broad spectrum of proteases without the direct blockage of the protease active site [17]. This can be considered as a manifestation of the decline associated with the use of this antiprotease during repair processes occurring in mucous membranes damaged by the inflammatory process. Also, when Wong and Saha investigated the acute phase response in patients with pulmonary tuberculosis, they showed a decrease in the concentration of A2M under treatment [18]. Anabalgan and Sadique also suggested that the decrease in serum A2M concentration was related to the effect of anti-inflammatory drugs, which reduced the level of prostaglandins responsible for A2M synthesis [19]. However, due to the slight increase in inflammation, there is no induction of additional synthesis of this protein [20, 21]. Again, no similar data for children was found in the literature.

During and because of the treatment, the inflammation in pseudocroup was relatively quickly reduced, which was followed by the significant difference between the APP intensities in the first and third time points. This applies to the initially increased AGP concentration and inflammatory W2 and W3 variants as well as the ACT-RC (ACT reactivity coefficient). The remaining APPs were only slightly altered so that the implemented treatment and recovery did not cause significant changes of their concentration [22].

In our survey, the intensity of the inflammatory reaction during pseudocroup was higher in girls, resulting in an increased concentration of AGP and the W2 variant. In boys, differences in relation to the control group indicated a lower intensity of the inflammatory reaction (lower total AGP concentration; also in the 3rd time point study, where the difference with girls became significant, *Z* = −2.24; *p* = 0.025) and a shift of glycosylation towards a chronic process, resulting in a decrease of the W1, W2, and W3 variants.

## 5. Conclusions

1. The inflammatory reaction observed in children during subglottic laryngitis is of low intensity, and the glycosylation profile of the APP suggests a chronic inflammatory process
2. In a follow-up investigation (at least 3 weeks after the end of treatment), no normalization of the evaluated parameters was noted, but signs of persistence of the inflammatory process were observed

## Figures and Tables

**Table 1:** Results of the routine investigations for the study group (children suffering from pseudocroup only).

Parameter	The study group at the time of admission Me (Q25-Q75)	Reference values
Age (months)	37 (22-68)	*X*
WBC (×10^9^/l)	9.75 (7.20-11.70)	2-6 years: 4.5-13.0 7-12 years: 4.0-12.00
RBC (×10^12^/l)	4.47 (4.31-4.75)	2-6 years: 4.3-5.5 7-12 years: 4.5-5.5
HGB (g/dl)	12.3 (11.8-13.0)	2-6 years: 10.9-14.2 7-12 years: 12.0-15.5
HCT (%)	35.3 (33.7-37.1)	2-6 years: 34-41 7-12 years: 37-43
PLT (×10^9^/l)	311 (277-361)	2-6 years: 130-350 7-12 years: 130-350

**Table 2:** Results of the investigations of acute phase protein (APP) concentrations and glycosylation profiles for the control group and study group at the 3 time points (statistically significant differences are marked in bold; the Mann–Whitney *U* test value and *p* value are given).

APP	Control group (*n* = 22)	Sample 1 Me (Q25-Q75)	Difference with the control group	Sample 2 Me (Q25-Q75)	Difference with the control group	Sample 3 Me (Q25-Q75)	Difference with the control group
CRP	0 (0)	0 (0-3)	1.37 0.17	0 (0)	-0.43 0.67	0 (0)	0.04 0.97
AGP	870 (630-1000)	950 (720- 1230)	0.99 0.32	985 (800-1200)	1.39 0.16	785 (570-990)	-1.19 0.24
**AGP-W0%**	50.0 (45.4-52.5)	44.9 (41.4-48.5)	-2.82 0.005	**46.3 (42.2-49.6)**	**-2.27** **0.02**	47.9 (42.3-51.0)	-1.19 0.23
**AGP-W1%**	**42.7 (40.6-43.9)**	**38.8 (36.8-42.7)**	**-2.99** **0.003**	**39.3 (38.0- 42.7)**	**-2.71** **0.01**	**39.9 (37.0-42.5)**	**-2.90** **0.004**
**AGP-W2%**	**6.6 (5.5-8.0)**	**11.9 (8.4-15.0)**	**4.49** **0.00001**	**10.5 (8.6- 13.4)**	**4.41** **0.00**	**10.3 (7.3- 13.0)**	**3.74** **0.0002**
**AGP-W3%**	**0.6 (0-1.9)**	**3.3 (1.9-5.3)**	**3.76** **0.0002**	**2.8 (0-5)**	**2.77** **0.01**	2.2 (0-5.2)	1.68 0.09
**AGP-RC**	1.0 (0.9-1.2)	1.2 (1.1-1.4)	2.83; 0.005	**1.2 (1.0-1.4)**	**2.24** **0.025**	1.1 (1.0-1.4)	1.24 0.22
ACT	340 (290-400)	380 (290-470)	1.03 0.303	390 (310-550)	1.42 0.16	313 (240-350)	-1.82 0.07
ACT-A1%	22.5 (19.4-25.8)	22.6 (18.5-25.5)	-0.73 0.467	23.1 (19.1-25.7)	-0.31 0.76	24.9 (23.5-27.0)	1.27 0.21
**ACT-A2%**	**31.8 (29.2- 34.3)**	**29.1 (25.9- 31.3)**	**-2.51** **0.012**	29.8 (26.8- 31.7)	-1.71 0.09	30.3 (27.1- 31.9)	-1.70 0.09
ACT-A3%	22.6 (15.8- 24.6)	22.5 (18.5-29.4)	1.37 0.171	22.3 (18.3- 28.0)	0.83 0.41	20.9 (17.3- 26.6)	0.42 0.67
**ACT-A4%**	20.8 (18.4- 25.6)	18.2 (12.9- 20.9)	-1.98 0.047	17.1 (11.3- 23.2)	-1.89 0.06	**12.9 (9.1- 19.3)**	**-3.39** **0.001**
**ACT-A5%**	2.9 (0-4.4)	4.8 (0-8.9)	1.76 0.08	**5.1 (3.9-7.6)**	**2.20** **0.03**	**6.9 (4.0-12.0)**	**3.63** **0.001**
ACT-RC	3.5 (2.9- 4.2)	3.4 (2.9- 4.4)	0.72 0.474	3.3 (2.9- 4.3)	0.32 0.75	3.0 (2.7- 3.3)	-1.28 0.201
**Tf**	**2725 (2270- 3040)**	**2240 (1810- 2700)**	**-2.68** **0.007**	**2295 (1830- 2720)**	**-2.37** **0.018**	**2220 (1810- 2720)**	**-2.375** **0.018**
**Tf-T1%**	4.0 (3.4–6.2)	**5.9 (4.6-7.8)**	**2.43** **0.015**	5.6 (3.9-7.6)	1.50 0.13	5.4 (4.0-6.6)	1.24 0.24
Tf-T2%	14.3 (12.7–18.9)	**18.6 (15.2-21.1)**	**2.52** **0.012**	**18.3 (16.7-21.2)**	**2.84** **0.004**	18.7 (14.3-22.2)	1.97 0.05
Tf-T3%	73.4 (62.5-78.3)	70.5 (62.1-74.0)	-1.33 0.18	70.6 (65.7-73.6)	-0.95 0.34	71.3 (65.9-76.1)	-0.52 0.61
**Tf-T4%**	**5.7 (4.6-13.9)**	**3.9 (2.3-7.8)**	**-2.77** **0.001**	**3.2 (2.2-6.4)**	**-3.58** **0.0003**	**3.2 (2.2-7.8)**	**-2.74** **0.01**
**A2M**	5.2 (4.9-5.6)	5.0 (3.8-5.6)	-1.72 0.085	**4.8 (4.0-5.5)**	**-2.10** **0.034**	4.9 (4.1-5.4)	-1.80 0.072
Cp	455 (400-540)	510 (380-620)	1.01 0.312	477 (390-542)	0.26 0.797	489 (430-600)	1.24 0.213
Hp	1.0 (0.8-1.3)	0.9 (0.7-1.4)	-0.32 0.745	1.2 (0.8-1.5)	1.32 0.187	**0.7 (0.4-1.1)**	**-2.54** **0.011**
AT	2.0 (1.9-2.1)	1.8 (1.5-2.2)	-1.86 0.063	1.8 (1.6-2.2)	-1.69 0.089	**1.6 (1.3-2.0)**	**-3.41** **0.001**

**Table 3:** Results of the statistical comparison of the results obtained at three time points (sample nos. 1, 2, and 3) investigated with the use of the Wilcoxon test for related variables (statistically significant differences are marked in bold; the *Z* test value and *p* value are given; ns—not significant).

APP	Difference between sample nos. 1 and 2	Difference between sample nos. 1 and 3	Difference between sample nos. 2 and 3
CRP	ns	ns	ns
**AGP**	**ns**	**3.04; 0.002**	**3.33; 0.001**
**W0%**	**ns**	**2.08; 0.037**	ns
W1%	ns	ns	ns
**W2%**	**ns**	**2.44; 0.014**	ns
**W3%**	**ns**	**2.44; 0.014**	ns
AGP-RC	ns	ns	ns
**ACT**	**ns**	**3.34; 0.004**	**2.83; 0.005**
**A1%**	**ns**	**2.83; 0.005**	**2.64; 0.008**
A2%	ns	ns	ns
**A3%**	**ns**	**2.71; 0.007**	ns
A4%	ns	ns	ns
A5%	ns	ns	ns
**ACT-RC**	ns	**2.83; 0.005**	**2.33; 0.020**
Tf	ns	ns	ns
T1%	ns	ns	ns
T2%	ns	ns	ns
T3%	ns	ns	ns
T4%	ns	ns	ns
A2M	ns	ns	ns
Cp	ns	ns	ns
**Hp**	**2.78; 0.005**	**3.06; 0.002**	**3.76; 0.0001**
AT	ns	ns	ns

## Data Availability

The data used to support the findings of this study are available from the corresponding author upon request.
